# How Glyphosate Impairs Liver Condition in the Field Lizard* Podarcis siculus* (Rafinesque-Schmaltz, 1810): Histological and Molecular Evidence

**DOI:** 10.1155/2019/4746283

**Published:** 2019-05-14

**Authors:** Mariailaria Verderame, Rosaria Scudiero

**Affiliations:** Department of Biology, University Federico II, Via Cintia, 80126 Napoli, Italy

## Abstract

The potential toxicity of glyphosate, a widely used broad-spectrum herbicide, is currently a great matter of debate. As vertebrate insectivores, lizards protect plants from herbivorous insects increasing plant biomass via the trophic cascade and represent an important link between invertebrates and higher predators. A negative effect of glyphosate on lizards' survival could have major impacts at the ecological levels. In this study, we investigated the effects of the exposure to low doses of glyphosate on the liver of the wall lizard* Podarcis siculus,* a suitable bioindicator of soil pollution. Two different doses of pure glyphosate (0.05 and 0.5 *μ*g/kg body weight) were orally administered every other day for 3 weeks to sexually mature males and females. The results demonstrated that both doses, despite being very low, are toxic for the liver that showed clear signs of suffering, regardless of sex. The histological analysis provided a scenario of severe hepatic condition, which degenerated until the appearance of fibrotic formations. The morphological observations were consistent with a loss of liver physiological functions. Immunocytochemical investigations allowed us to detect an involvement of antioxidant/cytoprotective proteins, such as superoxide dismutase 1 (Cu/Zn SOD, known as SOD1), glutathione peroxidase 1 (GPx1), metallothionein (MT), and tumor suppressor protein 53, (p53) suggesting that the liver was trying to react against stress signals and damage induced by glyphosate. Finally,* in situ* hybridization and Real-Time PCR analysis showed the upregulation of estrogen receptor *α* and vitellogenin gene expression, thus demonstrating the xenoestrogenic action of glyphosate. The imbalance of the hormonal homeostasis could threaten the lizards' reproductive fitness and survival, altering the trophic cascade.

## 1. Introduction

During the last two decades, environmental pollution caused by the intense use of herbicides is becoming an outstanding problem that can affect human and animal health through feeding [1, 2]. Recently, more attention has been paid to the potential toxicity induced by glyphosate (Gly) (N-[phosphonomethyl] glycine, C_3_H_8_NO_5_P) and its commercial formulations [2, 3]. Glyphosate-based herbicides (GBHs) are broad spectrum, nonselective, and nonsystemic herbicides widely used in agricultural and nonagricultural systems [4]. In addition to their use as weed-control herbicides, GBHs are also used as desiccants prior to harvest to accelerate natural drying of seeds. Gly herbicidal action derives from the inhibition of the enolpyruvylshikimate-3-phosphate synthase (EPSPS), a key plant enzyme involved in the synthesis of aromatic amino acids essential for plants growth and for the activation of defence mechanisms in vegetal cells [5]. Since this enzyme is absent in animals, it has long been assumed that glyphosate would not affect non-target species and, today, this is a great matter of debate. Classified by the United States Environmental Protection Agency (EPA) as “nontoxic and not an irritant” and by the European Food Safety Authority (EFSA) as “not carcinogenic to humans,” multiple lines of evidence suggest that GBHs pose a serious health risk to wildlife [6–15]. Over time, attention has been initially given to the Gly or GBHs adverse effects on aquatic organisms; Gly-exposed fish show hepatic damages, alterations in antioxidant enzyme systems, interference with steroid hormones, and increased level of plasma glucose and cortisol [7, 8]. Investigations on terrestrial nonmammalian vertebrates are scarce and mainly related to GBHs. In amphibians, GBHs cause teratogenic effects and developmental failures impacting both larval and adult stages [3, 9, 10]. In reptiles, several studies demonstrate a significant increase in DNA damage and alterations in the immune parameters, in plasma proteins, and in growth after exposure to GBHs [11–14]. High rates of crossed beaks, keratin disorder, and morphophysiological alterations of the male genital system have been also observed in GBHs-exposed birds [15]. The association between exposures to GBHs and health effects has been evaluated also in humans, including carcinogenic potential [2, 16, 17]. However, many studies reported controversial results on Gly effects, especially in humans [18]; in addition, most of them focused on the toxicity of the commercial formulations of Gly, such as the Roundup® (Monsanto), but the adjuvants or surfactants can often enhance the Gly toxic effects [19].

The work described here aimed to elucidate the effect of pure glyphosate on liver of terrestrial vertebrates, the organ most involved in detoxification. The experimental model was the Italian wall lizard* Podarcis siculus*, widely used as model organism for ecotoxicological analysis concerning soil contamination [20–22].

By using a multidisciplinary approach, we examined liver morphology and the appearance or changes in biomarkers of cell stress and injuries in lizards exposed by oral gavage to two low doses of pure Gly. The xenoestrogenic effect of this substance was also evaluated.

## 2. Materials and Methods

### 2.1. Animals and Experimental Design

*Podarcis siculus* specimens were captured by noose or hand near Gragnano, in a natural protect area of the Regional Park of Monti Lattari (Campania, Italy) during the postreproductive period (October). Only sexually mature specimens were considered [23]; the biometrical features were as follows: males, snout to vent length (SVL) of 69.3 ± 8 mm and 8.5 ± 1.2 g of body weight (bw); females, SVL of 58.2 ± 6 mm, 7.8 ± 0.9 bw. The lizards were randomly assigned to three groups; each group (n=10) was housed in its own terrarium (115 cm × 35 cm × 48 cm) that was covered with sand on the bottom and contained hiding places, such as hollow bricks. The three resulting terraria were maintained at 22 ± 2°C and exposed to the natural photoperiodic conditions. Live mealworms were used to feed lizards, and water was accessed freely. After 7 days of acclimatization, lizards of groups 1 and 2 were administered pure Gly at doses of 0.05 and 0.5 *μ*g/kg bw in 50 *μ*l tap water, respectively, via oral gavage every other day for 3 weeks; animals of group 3 (control) received by gavage the same doses of tap water. Animals were exposed to pure Gly to exclude possible interference of the adjuvants. These experimental doses correspond to 10^4^x and 10^3^x Acceptable Daily Intake (ADI), relative to the European ADI in mammals [24]. They also correspond to aqueous solutions of 0.1 and 1 *μ*g/L, respectively. Considering that Gly is generally sprayed at the concentration of 3.6 g/L, the doses used for this study should be considered low; it is conceivable that wild animals such as lizards can ingest equal or greater amounts of glyphosate.

At the end of treatments, animals were killed by decapitation after deep anaesthesia with ketamine hydrochloride (Parke-Davis, Berlin, Germany), 325 *μ*g/g bw; livers were immediately excised and processed for histological and biomolecular analysis.

The experiments were carried out following the ethical provisions established by the 2010/63/EU directive for animal experiment and were approved by the Ethical Committee for Animal Experiments, University of Naples Federico II (ID: 2013/0096988), according to the Italian law. They were organized to minimize stress and number of animals used.

### 2.2. Light Microscopy

Liver sections were obtained as previously described [22]. Some sections were stained for morphological analysis, and others were processed as reported below.

### 2.3. Melanin Bleach and Lipofuscin-Sudan Black Staining

For melanin detection in liver cells, dewaxed and rehydrated sections were incubated in 0.3% potassium permanganate solution containing 0.3% sulphuric acid for 2 h, according to the procedure described by Sheehan and Hrapchak [25]. Twin sections for the bleach control were in distilled water until the following step. Subsequently, all the sections were incubated in 1% oxalic acid until colourless, washed in tap water, and stained with ematoxylin-eosin.

Lipofuscin is an aggregate of oxidized proteins that accumulates progressively in aged cells. Lipofuscin staining is considered a powerful biomarker of stress-induced cellular senescence [26]. For lipofuscin revelation in lizard hepatocytes, twin sections, dewaxed and rehydrated, were treated o/n with Sudan Black B saturated solution in 70% ethanol. The sections were then rinsed in 70% ethanol until background became pale gray, washed in tap water, and mounted using an aqueous mountant.

### 2.4. Periodic Acid-Schiff Staining with Diastase

Periodic Acid-Schiff staining with Diastase was performed according to the protocol described by Mazzi [27]. Rehydrated liver slides were placed in *α*-amylase (diastase) solution (1%) for 20 min. Amylase-treated slides were immersed in 0.5% periodic acid for 10 min and then in Schiff's reagent for 15 min. Sections were washed in running lukewarm tap water for 5–10 min in order to develop a pink colour and then were counterstained in hematoxylin. Duplicate sections were processed as described above but with omitting amylase treatment.

### 2.5. Immunohistochemistry

For the immunostaining, liver sections were dewaxed, rehydrated, and processed by using the Novolink Max Polymer Detection System (Leica Biosystems, Nussloch, Germany), as previously described [28]. The antibodies used were as follows: anti-SOD1 (Thermo Fisher), anti-GPx1 (Thermo Fisher), anti-p53 (Thermo Fisher), anti-MT (Abcam), and anti-type IV collagen (SantaCruz Biotechnology), all diluted 1:200.

### 2.6. *In Situ* Hybridization

For* in situ* hybridization analysis, cDNA fragments encoding* ERα*,* ERβ,* and* VTG* were used [22]. Dig-labeled cDNA probes were generated by PCR using the DIG High Prime DNA labeling kit (Roche Diagnostics) and used at a concentration of 80ng/100*μ*L, as previously described [29].* ERα*,* ERβ,* and VTG immunolocalization were performed only on livers from male specimens, because they are more sensitive to xenoestrogenic substances; in addition, the use of males avoids the risk that the responses are perturbed by circulating estrogens.

### 2.7. RNA Purification and cDNA Synthesis

Total RNA and the following cDNA synthesis from each lizard liver were performed as described [30].

### 2.8. Quantitative Real-Time PCR Analysis

The Real-Time PCR reactions were carried out in triplicate for each sample, as described [26]. Specific primers for* P. siculus ERα, ERβ, *and* β-actin* genes were designed as previously reported [22, 31]. Once again,* ERα* and* ERβ *expression analyses were performed only on livers from male specimens.

### 2.9. Statistical Analysis

Data are presented as mean ± standard error of the mean (s.e.m.) from three separate experiments of each sample. Statistical analyses were carried out by GraphPad Prism 7 software. All the animals used for this study were captured in the same site and the body mass and SVL of male specimens used for the statistical analysis were comparable. For these reasons, the differences between the mean values were analysed by one-way analysis of variance (ANOVA), followed by Fisher's LSD test. The differences were considered significant when p<0.05.

## 3. Results

### 3.1. Hematoxylin-Eosin and Mallory's Trichrome

Hematoxylin-eosin stain allowed us to verify the general condition of the liver and Mallory's trichrome stain was used to show the presence of connective tissue.

**Figure 1:**
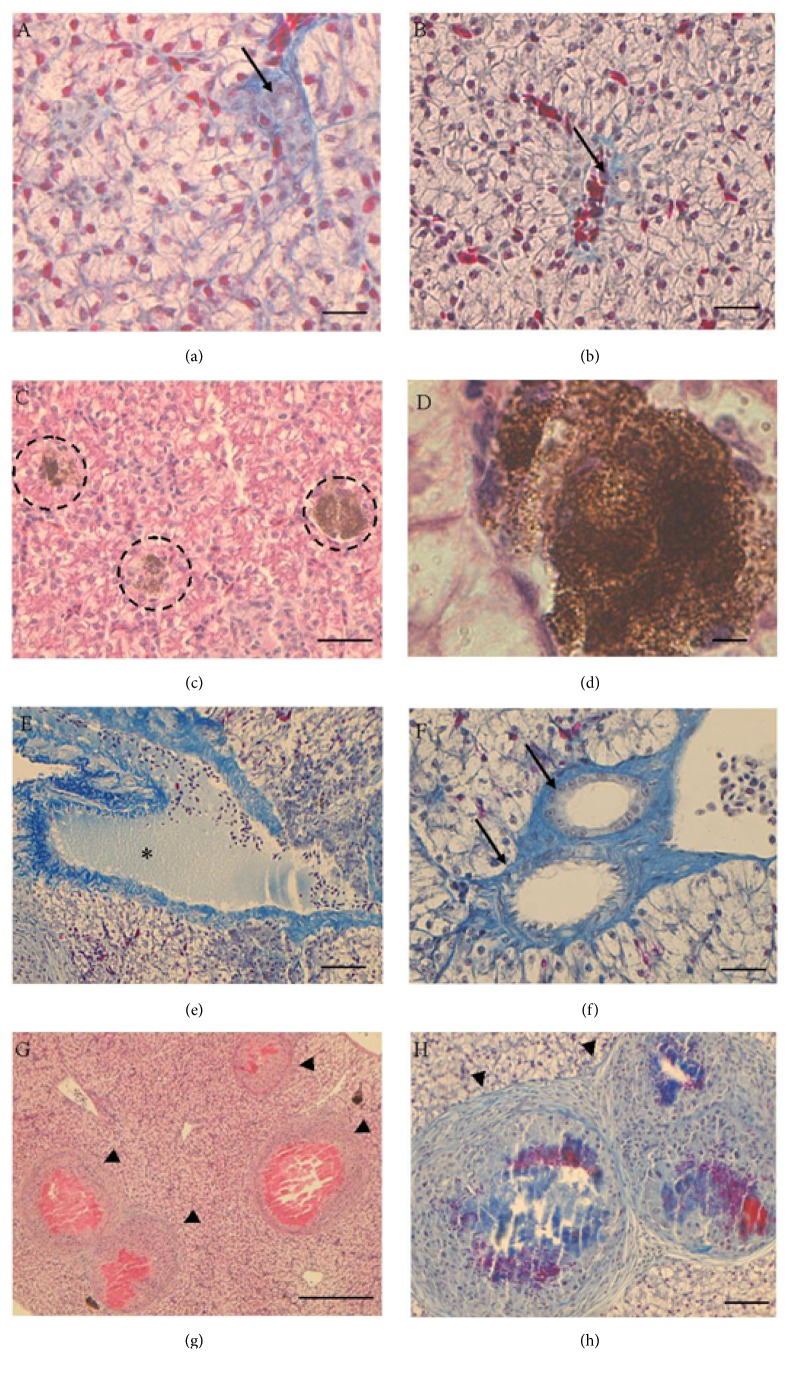
Morphological condition of liver. (a) Untreated lizard. (b) Lizards exposed to 0.05 *μ*g/kg Gly. (c–h) Lizards exposed to 0.5 *μ*g/kg Gly. Bile ducts (↑); degranulation areas (*◌*); blood vessel (*∗*); nodular/fibrotic structures (*◄*). (a, b, e, f, h) Mallory's trichrome; (c, d, g) hematoxylin-eosin. The bar is 30 *μ*m.

In untreated animals, the liver structure was regular, intrahepatic blood vessels were normal, and bile ducts were evident (Figure 1(a)). In the animals receiving lower Gly dose, liver parenchyma was characterized by the presence of few degranulation areas, whereas intrahepatic blood vessels and bile ducts were regular (Figure 1(b)).

In the animals receiving the higher Gly dose, the liver parenchyma appeared more damaged: large degranulation areas (Figures 1(c) and 1(d)) and swelling of blood vessels and bile ducts (Figures 1(e) and 1(f)) were evident. Nodular/cystic formations consisting of abundant connective fibers were also present in the hepatic tissue (Figures 1(g) and 1(h)).

### 3.2. Pigment Composition

To assess the composition of pale brown granules found in Gly-treated livers, melanin blanch and lipofuscin-Sudan Black staining were carried out.

**Figure 2:**
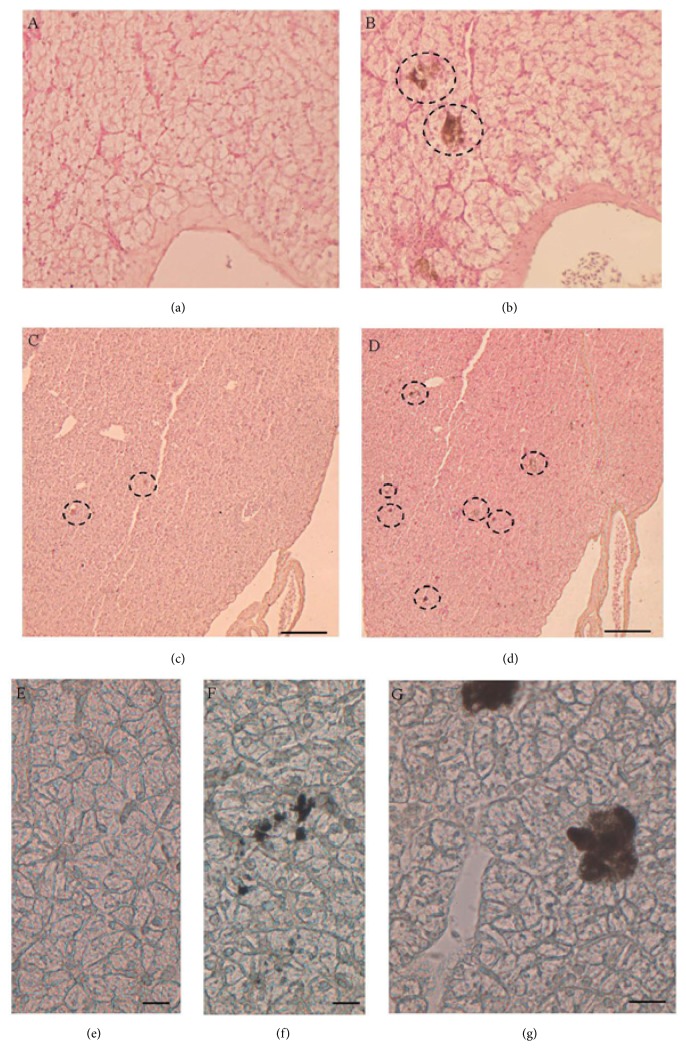
Melanin blanch and lipofuscin-Sudan black stain in liver. (a–d) Melanin blanch: in liver of 0.05 *μ*g/kg bw (a) and 0.5 *μ*g/kg bw (c) Gly-treated lizards the blanching of almost all granules is evident; in reaction control (b, d) the degranulation areas (*◌*) are evident. (e–g) lipofuscin-Sudan.

Melanin blanch reaction caused the blanching of almost all granules in the treated samples (Figures 2(a) and 2(c)); however few spots were yet evident (Figure 2(c)). In non-bleach control twin sections, obtained by omitting incubation with potassium permanganate, degranulation areas were still evident (Figures 2(b) and 2(d)).

Sudan Black stained sections showed that in the liver of Gly-treated animals, small spot granules were markedly Sudanophilic (Figures 2(f) and 2(g)); no colour appeared in untreated animals (Figure 2(e)).

### 3.3. Periodic Acid-Schiff (PAS) and PAS with Diastase

**Figure 3:**
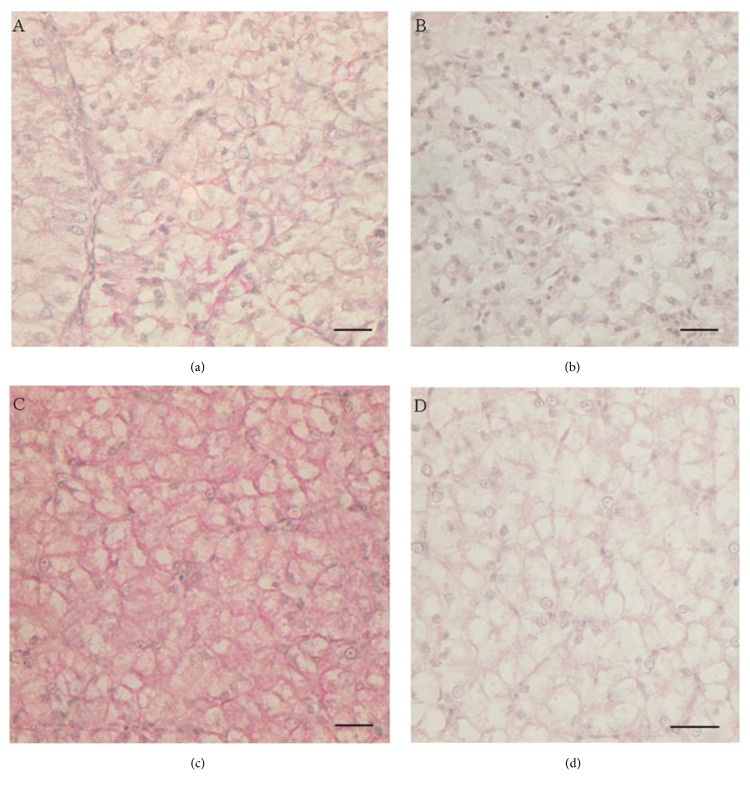
Periodic Acid-Schiff (PAS) and PAS with Diastase (PAS-D) stains in liver. (a, b) untreated animals: (a) PAS, (b) PAS-D. (c, d) Gly-treated lizards: (c) PAS, (d) PAS-D. Polysaccharides increase is evident in liver sections from lizards exposed to 0.05 *μ*g/kg bw Gly (c) with respect to untreated liver (a). A reduced staining is observed after diastase treatment ((b), untreated liver; (d), 0.05 *μ*g/kg bw Gly-treated liver). The staining does not change in liver sections from higher dose Gly-treated lizards (data not shown). The bar is 30 *μ*m.

PAS stain of liver sections showed an increase of polysaccharides in treated samples (Figure 3). In particular, the PAS-D stain (i.e., PAS reaction with predigestion of glycogen with the enzyme diastase) demonstrated an increase in liver glycogen in all Gly-treated samples (Figures 3(c) and 3(d)), no matter the dose (Figure 3).

### 3.4. Gly Effects on SOD1, GPx1, p53, MT, and Type IV Collagen Localization

Immunohistochemical analyses with SOD1, GPx1, p53, MT, and type IV collagen antibodies were performed to evaluate the ability of Gly to induce oxidative stress and cellular responses.

**Figure 4:**
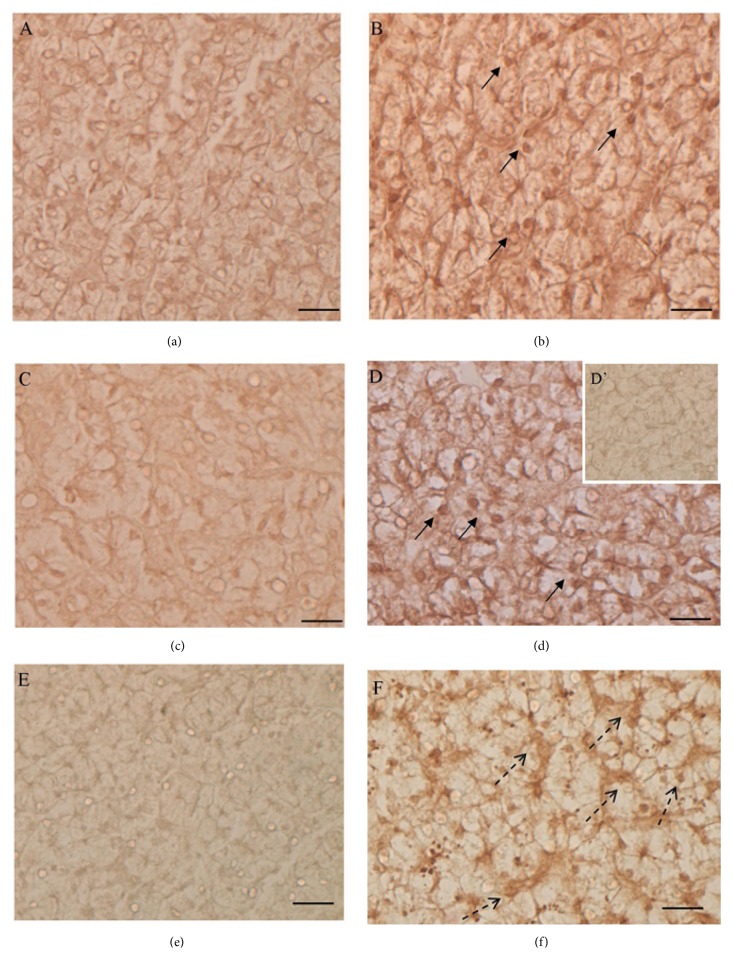
SOD1, GPx1, and p53 localization in lizard liver. SOD1 (a, b) and GPx1 (c, d) localization. In untreated lizards SOD1 (a) and GPx1 (c) are present in hepatocytes cytoplasm; in 0.05 *μ*g/kg bw Gly-treated lizards, SOD1 (b) and GPx1 (d) are detectable in the hepatocytes nuclei (↑) as well as in the cytoplasm. (e, f) p53 detection. The protein is absent in untreated animals (e) and detectable in the Kupffer cells (dotted arrow) of 0.05 *μ*g/kg bw Gly-treated lizards (f). (D'): negative control of reaction. Proteins localization does not change in 0.5 *μ*g/kg bw Gly-treated lizards (data not shown). The bar is 30 *μ*m.

In untreated lizards, SOD1 and GPx1 were mainly detected in the hepatocytes cytoplasm (Figures 4(a) and 4(c)). In the liver of Gly-treated lizards, both SOD1 (Figure 4(b)) and GPx1 (Figure 4(d)) were present also in nuclei.

Regarding p53, no immunohistochemical signal was present in the control animals (Figure 4(e)); after Gly treatments the p53 protein was detected in the Kupffer cells and in some hepatocytes nuclei (Figure 4(f)).

**Figure 5:**
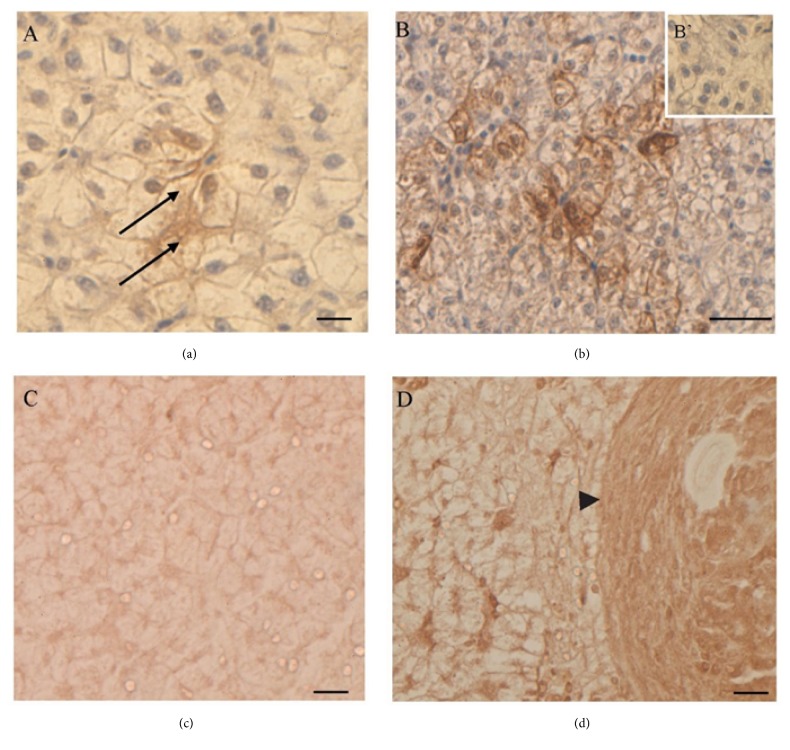
MT and type IV collagen localization in lizard liver. (a, b) MT localization: in untreated animals (a) the protein is evident in Kupffer cells (↑); in 0.5 *μ*g/kg bw Gly-treated animals (b), MT is evident in the cytoplasm of some clusters of hepatocytes. (c, d) Type IV collagen detection: in untreated animals (c) slight positivity is evident in the liver; in 0.5 *μ*g/kg bw Gly-treated animals, nodular/fibrotic structures are strongly positive to the antibody (*◄*). (B'): control of reaction. Proteins localization does not change in 0.05 *μ*g/kg bw Gly-treated lizards (data not shown). The bar is 30 *μ*m.

In control livers, MT immunolocalization demonstrated the presence of this protein mostly in Kupffer cells, as previously described [32] (Figure 5(a)). In Gly-treated livers, MT was also synthesized in the cytoplasm of some clusters of hepatocytes (Figure 5(b)).

### 3.5. *ERα*,* ERβ,* and* VTG* mRNA Expression in Liver

A possible estrogen-like action of glyphosate in the two different experimental conditions was evaluated, assaying* ERα*,* ERβ,* and* VTG* mRNA presence/abundance in male lizard liver by using* in situ* hybridization and Real-Time PCR analyses.

**Figure 6:**
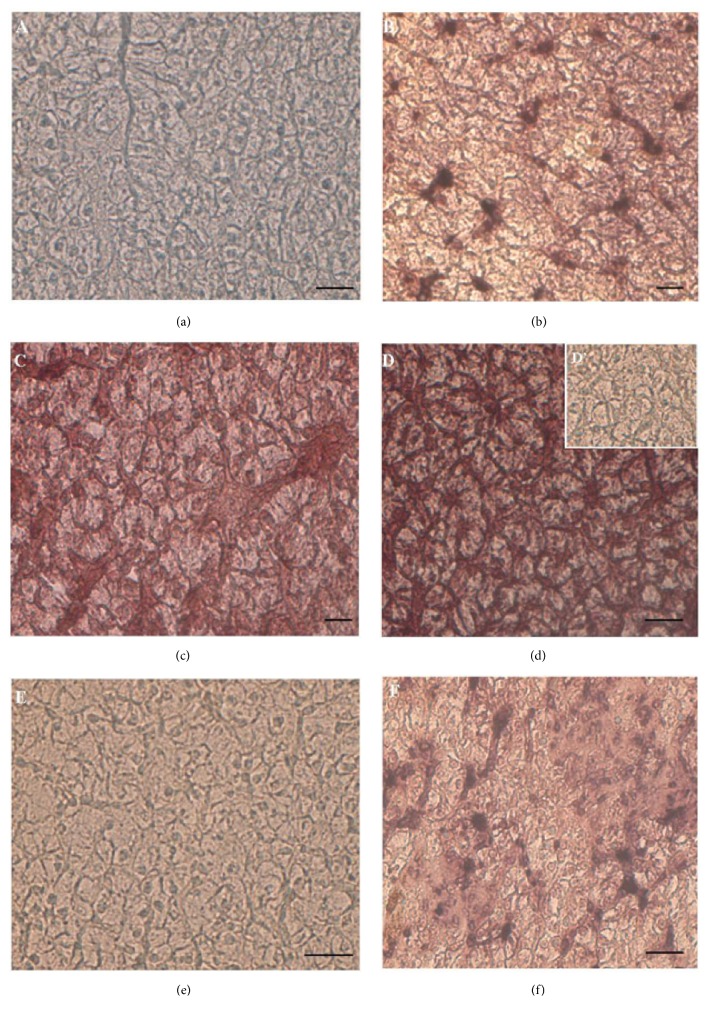
*ERα*,* ERβ,* and* VTG* mRNA localization in male liver. (a, c, e) untreated lizards: neither* ERα* (a) nor* VTG* (e) transcripts are detectable;* ERβ* mRNA (c) is evident in the hepatocytes. (b, d, f) 0.05 *μ*g/kg bw Gly-treated lizards:* ERα* (b),* ERβ* (d), and* VTG* (f) mRNAs are evident in the liver samples. (D'): control of reaction. mRNA localization does not change in lizards treated with 0.5 *μ*g/kg bw Gly (data not shown). The bar is 30 *μ*m.

In the liver of control animals, no positivity to* ERα* (Figure 6(a)) or* VTG* (Figure 6(e)) was evident, whereas* ERβ* transcripts were detected in males (Figure 6(c)), as expected [31]. In Gly-treated animals, liver sections showed the presence of both* ERα* (Figure 6(b)) and* VTG* (Figure 6(f)) transcripts, together with* ERβ* mRNA, being always evident (Figure 6(d)), no matter the Gly dose.

No signal was observed in negative control sections incubated by omitting* ERα*,* ERβ,* or* VTG* cDNA probes (Figure 6(D')).

**Figure 7:**
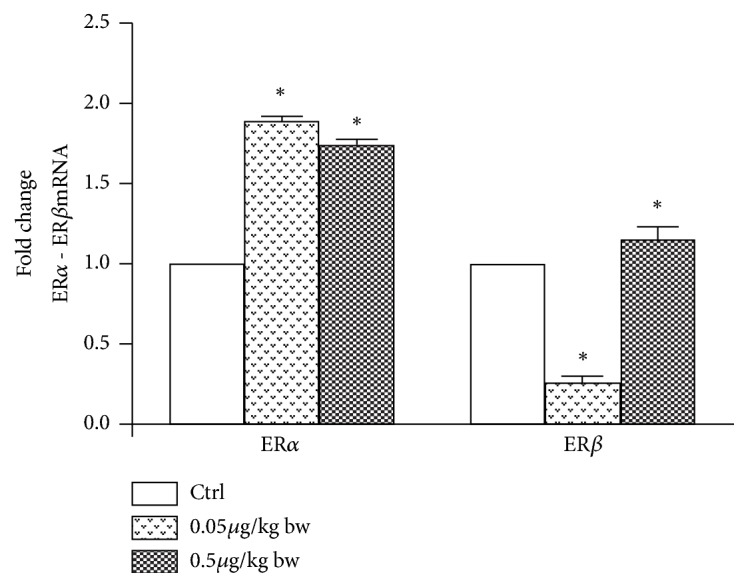
Effects of Gly exposure on* ERα* and* ERβ* expression in male lizard liver. The* ERα* and* ERβ* mRNAs expression in Gly-treated lizards, determined by Real-Time PCR analysis, was normalized to that of* β-actin* mRNA and converted in fold change, compared with the untreated control lizards. The data represent the mean ± s.e.m. from three separate experiments of each sample. Significance of differences is shown. *∗*p<0.05.

The changes in* ERα* and* ERβ* presence after Gly treatments prompted us to investigate the* ERs* expression levels in male liver by means of Real-Time PCR analysis. Results demonstrated the presence of a low quantity of* ERα* transcripts in control livers, below the detection limit of the* in situ* analysis, and confirmed the increase of these transcripts in treated animals (about 1-fold), no matter the Gly dose. Conversely,* ERβ* expression level was downregulated (of about 3-fold) in the animals treated with the lower Gly dose and upregulated (0.8-fold) following the 0.5 *μ*g/kg Gly dose administration (Figure 7).

## 4. Discussion

The present study provides a further understanding of possible mechanisms of Gly toxicity in the liver, the first detoxification organ, particularly sensitive to dietary pollutants. Although a plethora of studies warned about the risks associated with the use of glyphosate or GBHs, whether the exposure to this herbicide causes health diseases to humans or animals is still a matter of debate [2, 33, 34]. Meantime, many countries allow Gly use to control invasive plant species; the European Commission in November 2017 authorised the Gly use for another 5 years.

The results described here provide first evidence of hepatic toxic effect of pure Gly on a terrestrial reptile. Indeed, previous investigations on reptiles focused only on GBH effects, mainly on marine and freshwater turtles [11, 35], embryos [12, 36], or adult immune system [13, 14]. Our data demonstrate that pure Gly administered orally is toxic and exerts a xenoestrogenic action in the liver of* Podarcis siculus* even at doses that can be considered low if compared to the Gly working solution generally used. In particular, a generalized increase in hepatic glycogen is observed, regardless of the dose, and degranulation areas constituted mostly by melanin and lipofuscin are present. An increase in degranulation areas and the swelling of blood vessels and bile ducts are evident in the lizards treated with the slightly higher Gly dose. Noteworthy, the presence of nodular/cystic formations made by connective tissue is also detected within the hepatic tissue following the treatment with the higher Gly dose. These formations could testify the onset of liver fibrosis, since they consist of abundant type IV collagen, one of the major markers of this disease [37].

All together, these results demonstrate a serious hepatic suffering and are in agreement with those retrieved by studies carried out in fish [38–40]. However, the results achieved in* P. siculus* liver are sex-independent, whereas in the fish* Poecilia reticulata* the Gly-induced hepatic diseases are more common in males [41].

Degranulation areas and lipofuscin depots could be ascribed to Gly-induced oxidative stress in liver, considering that the presence of melanin represents a cellular response to remove the free radicals [42]. Indeed, it is known that many environmental pollutants induce the formation of reactive oxygen species in cells; it has been demonstrated that Gly and GBHs also give rise to cell stress in fishes and amphibians [8, 43, 44]. In cells, the harmful effects of free radicals are balanced by the activity of antioxidant enzymes such as SOD1 and GPx1, together with the action of nonenzymatic antioxidants such as metallothioneins, small, cysteine-rich, heavy metal-binding proteins that are able to protect cells from oxidative stress [45, 46].

In this frame, interestingly enough are the changes observed in the cellular localization of antioxidant enzymes in liver of Gly-treated lizards. In particular, the nuclear localization of GPx-1 in Gly-treated hepatocytes might facilitate the antioxidant functions of this enzyme, as proposed for hepatic cancer cells [47]; also, the presence of nuclear SOD1 in these cells might be ascribed to its function as a guardian of the genome by scavenging superoxides generated into or near the nucleus [48].

Following Gly administration, it is possible to observe clusters of hepatocytes synthesizing MT, thus demonstrating that Gly induces a cellular response, which includes the nonenzymatic antioxidants pathway. These data are in agreement with those observed in rats, where the histopathological changes engendered by the herbicide ameliorated when the glyphosate exposure was preceded by a zinc supplementation, which was able to induce a massive MT synthesis [49].

Moreover, the liver fighting against stress signals induced by Gly is also demonstrated by the activation of p53 in Kupffer cells. These cells are specialized macrophages located in the liver and are considered critical mediators of both liver injury and repair [50]; the increase in p53 following Gly-induced stress signals could determine cell fate by specifically regulating cell cycle arrest, DNA repair, and apoptosis [51].

Finally, our data show that Gly exhibits a clear estrogenic activity, even at the low concentrations tested in this study. Indeed, males treated with Gly display the hepatic biosynthetic alterations typical of an estrogenic contamination, such as the transcription of both* VTG* and* ERα* gene in the liver. VTG is a liver protein synthesized under estrogen stimulation only in females of oviparous vertebrates during the reproductive period. Being induced by estrogen, VTG is widely recognized as a biomarker of xenoestrogenic pollution; in* P. siculus,* VTG transcription is strictly dependent on the ER*α* presence and this allowed the consideration of ER*α* also being a good indicator of environmental pollution in this species [22, 31, 52]. After Gly treatment, the* ERα* and* ERβ* expression patterns are different.* ERα* expression significantly increases following both Gly-treatments, whereas* ERβ* transcription is downregulated at lower Gly dose and upregulated of about 0.8-fold at higher dose. The* ER*-mediated action of Gly was demonstrated,* in vitro*, in human breast cancer cells, in which ERE transcription activity induced by Gly was inhibited by ICI 182780, an estrogen antagonist [53]. Therefore, the ability of Gly to switch on* VTG* and* ERα* genes transcription leads us to conclude that this herbicide holds a xenoestrogenic activity exerting the stimulatory effects via the ER-dependent pathway, interfering with the physiological estrogenic signalling. Since the possible mechanism of Gly-ERs interaction is still unknown, it could be hypothesized that Gly stimulates an indirect estrogenic activation pathway that leads, as a last step, to the activation of the estrogens responsive genes; however, this hypothesis needs to be further investigated.

It is also reported that nuclear SOD1 interacts with ER*α*, and this association seems to be required for effective activation of estrogen-responsive genes [54]. Then, in the light of what has been observed in lizard liver, it is possible to hypothesize that Gly determines the translocation of SOD1 in the nucleus that, in turn, elicits the activation of the genes responsive to the estrogens, such as VTG.

In conclusion, the overview of the results described here indicates that the oral administration of pure Gly at very low doses is able to induce hepatotoxic and estrogen-like action in the lizard* P. siculus*. Histological and biomolecular analysis showed a severe scenario of the hepatic condition, which degenerates until the appearance of fibrotic formations and the induction of strictly estrogen-dependent molecules. Nevertheless, the liver cells try to fend boosting up their defence mechanism alerting both enzymatic and nonenzymatic oxidative stress systems. In our opinion, the results we have documented are biologically relevant and could indicate endangering to the viability and survival of lizard populations, thus threatening plants and insects through the imbalance of the trophic cascade. Glyphosate is highly soluble in water and moderately persistent in the soil; it cannot be excluded that the lizards in their natural habitat come into contact with contaminated water and food. Further research is needed to measure potential genotoxic and reproductive impacts of glyphosate exposure on these reptiles; meanwhile, a drastic drop in the use of glyphosate would be desirable which, through the food chain, might bring serious risk for wildlife.

## Data Availability

The data used to support findings of this study are included within the article, with references being cited.

## References

[1] Acquavella J. F., Alexander B. H., Mandel J. S., Gustin C., Baker B., Chapman P. (2004). Glyphosate biomonitoring for farmers and their families: Results from the farm family exposure study. *Environmental Health Perspectives*.

[2] IARC Monographs (2015). *Some Organophosphate Insecticides and Herbicides*.

[3] Bach N. C., Marino D. J. G., Natale G. S., Somoza G. M. (2018). Effects of glyphosate and its commercial formulation, Roundup® Ultramax, on liver histology of tadpoles of the neotropical frog, *Leptodactylus latrans* (amphibia: Anura). *Chemosphere*.

[4] Baylis A. D. (2000). Why glyphosate is a global herbicide: Strengths, weaknesses and prospects. *Pest Management Science*.

[5] Dong Y., Ng E., Lu J. (2019). Desensitizing plant EPSP synthase to glyphosate: Optimized global sequence context accommodates a glycine-to-alanine change in the active site. *The Journal of Biological Chemistry*.

[6] Motta E. V. S., Raymann K., Moran N. A. (2018). Glyphosate perturbs the gut microbiota of honey bees. *Proceedings of the National Acadamy of Sciences of the United States of America*.

[7] Gholami-Seyedkolaei S. J., Mirvaghefi A., Farahmand H., Kosari A. A. (2013). Effect of a glyphosate-based herbicide in *Cyprinus carpio*: Assessment of acetylcholinesterase activity, hematological responses and serum biochemical parameters. *Ecotoxicology and Environmental Safety*.

[8] Webster T. M. U., Santos E. M. (2015). Global transcriptomic profiling demonstrates induction of oxidative stress and of compensatory cellular stress responses in brown trout exposed to glyphosate and Roundup. *BMC Genomics*.

[9] Paetow L. J., McLaughlin J. D., Pauli B. D., Marcogliese D. J. (2013). Mortality of American bullfrog tadpoles *Lithobates catesbeianus* infected by *Gyrodactylus jennyae* and experimentally exposed to *Batrachochytrium dendrobatidis*. *Journal of Aquatic Animal Health*.

[10] Wagner N., Reichenbecher W., Teichmann H., Tappeser B., Lötters S. (2013). Questions concerning the potential impact of glyphosate-based herbicides on amphibians. *Environmental Toxicology and Chemistry*.

[11] Héritier L., Duval D., Galinier R., Meistertzheim A.-L., Verneau O. (2017). Oxidative stress induced by glyphosate-based herbicide on freshwater turtles. *Environmental Toxicology and Chemistry*.

[12] Schaumburg L. G., Siroski P. A., Poletta G. L., Mudry M. D. (2016). Genotoxicity induced by Roundup® (Glyphosate) in tegu lizard (*Salvator merianae*) embryos. *Pesticide Biochemistry and Physiology*.

[13] Carpenter J. K., Monks J. M., Nelson N. (2016). The effect of two glyphosate formulations on a small, diurnal lizard (*Oligosoma polychroma*). *Ecotoxicology*.

[14] Latorre M. A., González E. C. L., Larriera A., Poletta G. L., Siroski P. A. (2013). Effects of *in vivo* exposure to Roundup® on immune system of *Caiman latirostris*. *Journal of Immunotoxicology*.

[15] Samsel A., Seneff S. (2013). Glyphosate’s suppression of cytochrome P450 enzymes and amino acid biosynthesis by the gut microbiome: pathways to modern diseases. *Entropy*.

[16] Andreotti G., Koutros S., Hofmann J. N. (2018). Glyphosate use and cancer incidence in the agricultural health study. *JNCI: Journal of the National Cancer Institute*.

[17] Van Bruggen A. H. C., He M. M., Shin K. (2018). Environmental and health effects of the herbicide glyphosate. *Science of the Total Environment*.

[18] Tarazona J. V., Court-Marques D., Tiramani M. (2017). Glyphosate toxicity and carcinogenicity: a review of the scientific basis of the European Union assessment and its differences with IARC. *Archives of Toxicology*.

[19] Vanlaeys A., Dubuisson F., Seralini G.-E., Travert C. (2018). Formulants of glyphosate-based herbicides have more deleterious impact than glyphosate on TM4 Sertoli cells. *Toxicology in Vitro*.

[20] Marsili L., Casini S., Mori G. (2009). The Italian wall lizard (*Podarcis sicula*) as a bioindicator of oil field activity. *Science of the Total Environment*.

[21] De Falco M., Sellitti A., Sciarrillo R. (2014). Nonylphenol effects on the HPA axis of the bioindicator vertebrate, *Podarcis sicula* lizard. *Chemosphere*.

[22] Verderame M., Limatola E., Scudiero R. (2016). Estrogenic contamination by manure fertilizer in organic farming: a case study with the lizard *Podarcis sicula*. *Ecotoxicology*.

[23] Zuffi M. A. L., Casu V., Marino S. (2012). The Italian wall lizard, *Podarcis siculus*, along the Tuscanian coast of central Italy: Biometrical features and phenotypic patterns. *The Herpetological Journal*.

[24] EFSA (2015). Conclusion on the peer review of the pesticide risk assessment of the active substance glyphosate. *EFSA Journal*.

[25] Sheehan D., Hrapchak B. (1980). *Theory and Practice of Histotechnology*.

[26] Georgakopoulou E. A., Tsimaratou K., Evangelou K. (2013). Specific lipofuscin staining as a novel biomarker to detect replicative and stress-induced senescence. A method applicable in cryo-preserved and archival tissues. *Aging*.

[27] Mazzi V. (1977). *Manuale di tecniche istologiche e istochimiche*.

[28] Verderame M., Limatola E., Scudiero R. (2017). Metallothionein expression and synthesis in the testis of the lizard *Podarcis Sicula* under natural conditions and following estrogenic exposure. *European Journal of Histochemistry*.

[29] Verderame M., Limatola E., Scudiero R. (2016). Ectopic synthesis of vitellogenin in testis and epididymis of estrogen-treated lizard *Podarcis sicula*. *General and Comparative Endocrinology*.

[30] Verderame M., Migliaccio V., Scudiero R. (2018). Role of estrogen receptors, P450 aromatase, PCNA and p53 in high-fat-induced impairment of spermatogenesis in rats. *Comptes Rendus Biologies*.

[31] Verderame M., Limatola E. (2010). Molecular identification of estrogen receptors (ER*α* and ER*β*) and their differential expression during VTG synthesis in the liver of lizard *Podarcis sicula*. *General and Comparative Endocrinology*.

[32] Simoniello P., Filosa S., Riggio M. (2010). Responses to cadmium intoxication in the liver of the wall lizard *Podarcis sicula*. *Comparative Biochemistry and Physiology - C Toxicology and Pharmacology*.

[33] Williams G. M., Aardema M., Acquavella J. (2016). A review of the carcinogenic potential of glyphosate by four independent expert panels and comparison to the IARC assessment. *Critical Reviews in Toxicology*.

[34] Williams G. M., Berry C., Burns M., de Camargo J. L. V., Greim H. (2016). Glyphosate rodent carcinogenicity bioassay expert panel review. *Critical Reviews in Toxicology*.

[35] Héritier L., Meistertzheim A.-L., Verneau O. (2017). Oxidative stress biomarkers in the Mediterranean pond turtle (*Mauremys leprosa*) reveal contrasted aquatic environments in Southern France. *Chemosphere*.

[36] López González E. C., Larriera A., Siroski P. A., Poletta G. L. (2017). Micronuclei and other nuclear abnormalities on *Caiman latirostris* (Broad-snouted caiman) hatchlings after embryonic exposure to different pesticide formulations. *Ecotoxicology and Environmental Safety*.

[37] Fallatah H. I. (2014). Noninvasive biomarkers of liver fibrosis: an overview. *Advances in Hepatology*.

[38] Topal A., Atamanalp M., Uçar A. (2015). Effects of glyphosate on juvenile rainbow trout (*Oncorhynchus mykiss*): Transcriptional and enzymatic analyses of antioxidant defence system, histopathological liver damage and swimming performance. *Ecotoxicology and Environmental Safety*.

[39] Braz-Mota S., Sadauskas-Henrique H., Duarte R. M., Val A. L., Almeida-Val V. M. F. (2015). Roundup® exposure promotes gills and liver impairments, DNA damage and inhibition of brain cholinergic activity in the Amazon teleost fish *Colossoma macropomum*. *Chemosphere*.

[40] dos Santos A. P. R., Rocha T. L., Borges C. L., Bailão A. M., de Almeida Soares C. M., de Sabóia-Morais S. M. T. (2017). A glyphosate-based herbicide induces histomorphological and protein expression changes in the liver of the female guppy *Poecilia reticulate*. *Chemosphere*.

[41] Antunes A. M., Rocha T. L., Pires F. S. (2017). Gender-specific histopathological response in guppies *Poecilia reticulata* exposed to glyphosate or its metabolite aminomethylphosphonic acid. *Journal of Applied Toxicology*.

[42] Rózanowska M., Sarna T., Land E. J., Truscott T. G. (1999). Free radical scavenging properties of melanin interaction of eu- and pheo-melanin models with reducing and oxidising radicals. *Free Radical Biology & Medicine*.

[43] Lushchak O. V., Kubrak O. I., Storey J. M., Storey K. B., Lushchak V. I. (2009). Low toxic herbicide Roundup induces mild oxidative stress in goldfish tissues. *Chemosphere*.

[44] Modesto K. A., Martinez C. B. R. (2010). Roundup® causes oxidative stress in liver and inhibits acetylcholinesterase in muscle and brain of the fish *Prochilodus lineatus*. *Chemosphere*.

[45] Ahmad I., Hamid T., Fatima M. (2000). Induction of hepatic antioxidants in freshwater catfish (*Channa punctatus Bloch*) is a biomarker of paper mill effluent exposure. *Biochimica et Biophysica Acta (BBA) - General Subjects*.

[46] Ruttkay-Nedecky B., Nejdl L., Gumulec J. (2013). The role of metallothionein in oxidative stress. *International Journal of Molecular Sciences*.

[47] Huang C., Ding G., Gu C. (2012). Decreased selenium-binding protein 1 enhances glutathione peroxidase 1 activity and downregulates HIF-1*α* to promote hepatocellular carcinoma invasiveness. *Clinical Cancer Research*.

[48] Inoue E., Tano K., Yoshii H. (2010). SOD1 is essential for the viability of DT40 cells and nuclear SOD1 functions as a guardian of genomic DNA. *Journal of Nucleic Acids*.

[49] Tizhe E. V., Ibrahim N. D.-G., Fatihu M. Y. (2014). Serum biochemical assessment of hepatic and renal functions of rats during oral exposure to glyphosate with zinc. *Comparative Clinical Pathology*.

[50] Dixon L. J., Barnes M., Tang H., Pritchard T. M. T., Nagy L. E. (2013). Kupffer cells in the liver. *Journal of Comparative Physiology*.

[51] Levine A. J., Oren M. (2009). The first 30 years of p53: growing ever more complex. *Nature Reviews Cancer*.

[52] Verderame M., Prisco M., Andreuccetti P., Aniello F., Limatola E. (2011). Experimentally nonylphenol-polluted diet induces the expression of silent genes VTG and ER*α* in the liver of male lizard *Podarcis sicula*. *Environmental Pollution*.

[53] Thongprakaisang S., Thiantanawat A., Rangkadilok N., Suriyo T., Satayavivad J. (2013). Glyphosate induces human breast cancer cells growth via estrogen receptors. *Food and Chemical Toxicology*.

[54] Rao A. K., Ziegler Y. S., McLeod I. X., Yates J. R., Nardulli A. M. (2008). Effects of Cu/Zn superoxide dismutase on estrogen responsiveness and oxidative stress in human breast cancer cells. *Molecular Endocrinology*.

